# Acetabular- and femoral orientation after periacetabular osteotomy as a predictor for outcome and osteoarthritis

**DOI:** 10.1186/s12891-020-03878-y

**Published:** 2020-12-26

**Authors:** Jens Goronzy, Lea Franken, Albrecht Hartmann, Falk Thielemann, Sophia Blum, Klaus-Peter Günther, Jörg Nowotny, Anne Postler

**Affiliations:** 1 University Center of Orthopedics and Traumatology, University Medicine Carl Gustav Carus Dresden, TU Dresden, Dresden, Germany; 2 Department of Radiology, University Medicine Carl Gustav Carus Dresden, TU Dresden, Dresden, Germany

**Keywords:** Periacetabular osteotomy, MRI, Acetabular version, Femoral torsion, McKibbi

## Abstract

**Background:**

Periacetabular osteotomy is a successful treatment for hip dysplasia. The results are influenced, however, by optimal positioning of the acetabular fragment, femoral head morphology and maybe even femoral version as well as combined anteversion have an impact. In order to obtain better insight on fragment placement, postoperative acetabular orientation and femoral morphology were evaluated in a midterm follow-up in regard to functional outcome and osteoarthritis progression.

**Methods:**

A follow-up examination with 49 prospectively documented patients (66 hips) after periacetabular osteotomy (PAO) was performed after 62.2 ± 18.6 months. Mean age of patients undergoing surgery was 26.7 ± 9.6 years, 40 (82%) of these patients were female. All patients were evaluated with an a.p. pelvic x-ray and an isotropic MRI in order to assess acetabular version, femoral head cover, alpha angle, femoral torsion and combined anteversion. The acetabular version was measured at the femoral head center as well as 0.5 cm below and 0.5 and 1 cm above the femoral head center and in addition seven modified acetabular sector angles were determined. Femoral torsion was assessed in an oblique view of the femoral neck. The combined acetabular and femoral version was calculated as well. To evaluate the clinical outcome the pre- and postoperative WOMAC score as well as postoperative Oxford Hip Score and Global Treatment Outcome were analyzed.

**Results:**

After PAO acetabular version at the femoral head center (31.4 ± 9.6°) was increased, the anterior cover at the 15 o’clock position (34.7 ± 15.4°) was reduced and both correlated significantly with progression of osteoarthritis, although not with the functional outcome. Combined acetabular and femoral torsion had no influence on the progression of osteoarthritis or outcome scores.

**Conclusion:**

Long-term results after PAO are dependent on good positioning of the acetabular fragment in all 3 planes. Next to a good lateral coverage a balanced horizontal alignment without iatrogenic pincer impingement due to acetabular retroversion, or insufficient coverage of the anterior femoral head is important.

## Background

Several mid- and long-term studies have shown periacetabular osteotomy (PAO) to be a successful treatment of hip dysplasia [1–3]. Increasing experience with this procedure and better understanding of hip pathomechanics not only helped defining a better patient selection but also improved the procedure itself. Over the years negative impact factors like advanced osteoarthritis (Kellgren and Lawrence ≥2), older age, higher body mass index and female sex could be determined. Additionally, the quality of the acetabular correction has an effect on the long-term survivorship. Since this operative modification is a complex 3-dimensional task not only acetabular lateral coverage but also acetabular anterior and posterior cover has an impact on mid and long-term outcome. It has been demonstrated that induced acetabular retroversion can lead to impingement causing progression of osteoarthritis and hip pain [4, 5]. A major limitation of all mentioned studies, however, is the lack of three-dimensional imaging for the assessment of acetabular version. Plain radiographs may be highly biased by pelvic tilt and other inherent limitations of conventional radiographs [6–8]. Magnet resonance imaging (MRI) has the capacity to provide accurate measurements without radiation exposure. To our knowledge no other study has used MRI in the follow-up of DDH patients treated with PAO. Further combined femoral and acetabular version may have an impact on pain and osteoarthritis progression. Some authors have described an association between abnormal combined anteversion and hip pain in unoperated patients [9].

The aim of this study was to obtain a better insight on the association of postoperative acetabular fragment placement and femoral influence. For this reason a detailed MRI assessment of hip geometry was correlated with patient reported outcome measures (PROMs) and postoperative progression of osteoarthritis (Table 1).

**Table 1:** Demographic description of the collective

	Preoperative		Postoperative	
Age	26,7 +/- 9,6 (13-46)			
BMI	23.5 ± 4.0 (172–35.6)			
Gender (female)	54 (81.8%)			
Side (left)	30 (45.5%)			
WOMAC	72.9 ± 19.3 (27.1–100)		91 ± 12.6 (45.8–100)	
Oxford Score	–		43.0 ± 6.2 (21–48)	
GTO	–		1.6 ± 0.8 (1–4)	
Ostearthritis (K&L)	0	57	0	38
	1	8	1	20
	2	1	2	7
	3	0	3	1
	4	0	4	0

Table 1 Demographic data of patients as well as outcome score presented with mean ± SD with range in parentheses and osteoarthritis progression classified by Kellgren and Lawrence (K&L).

## Methods

**Fig. 1:**
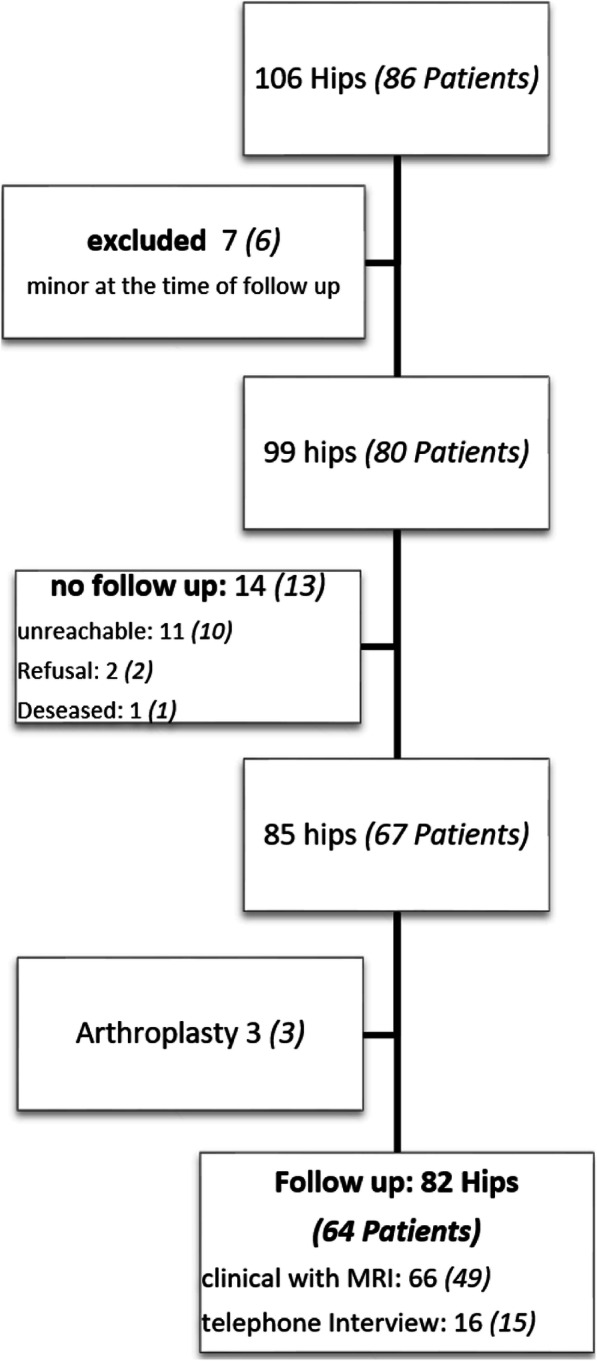
STROBE diagram. Diagram of operated hips (patients) and cases with completed follow-up

At our university center, isolated periacetabular osteotomy for hip dysplasia was performed on 106 hips (86 patients) from July 2005 to December 2010. After obtaining institutional approval of the ethic committee we performed a follow-up examination of 85 hips (67 patients). All 66 hips (49 patients) received the follow-up directly in our clinic using a pelvic and hip MRI with calculated radial sequences and an axial sequence of the knee condyles as well as a pelvic x-ray and frog leg view. This study focused on these patients exclusively (Fig. 1). The general follow-up time for these patients was 62.2 ± 18.6 (31–102) months consisting out of 54 female (40 patients) and 12 male (9 patients) hips. Mean age at the time of operation was 26.7 ± 9.6 years.

All surgical procedures were performed by one experienced surgeon (KPG). Patients were offered PAO when a decreased lateral center-edge (CE) angle was present. In addition hip pain, which did not respond adequately to conservative therapy, had to be present for at least 6 months. Contraindications for this procedure during the study period were advanced radiographic osteoarthritis (Kellgren & Lawrence Grade 3 and 4), incongruence of joint space on pelvic AP radiographs or abduction view, or patient age > 50 years. All patients were checked for head-neck offset alterations during operation with a capsulotomy and were corrected if needed.

Before intervention, as well as during follow-up, the WOMAC score was obtained. In addition, Oxford hip score and Global treatment outcome score (GTO) were measured during follow-up [10–12].

For image acquisition a standard three-dimensional proton density scan using Sampling Perfection with Application optimized Contrasts using different flip angle Evolution (SPACE) with an isotropic voxel of 0.9 mm, customized for optimal field of view and acquisition time, was obtained. For femoral torsion measurement an additional transverse T2 Haste Localizer of the knees was performed. A 1.5-T MRI Scanner (Siemens Somatom Avanto; Siemens HealthCare, Erlangen, Germany) was used. Since most patients were presented preoperatively with externally performed MRIs, no standardization and therefore no comparative analysis between pre- and postoperative images could be done.

**Fig. 2:**
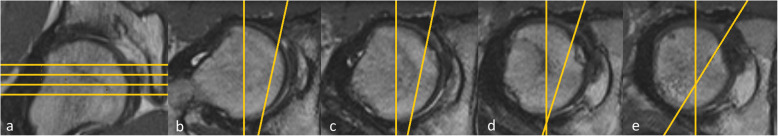
Acetabular Version. Measurement of acetabular version **a** at the level of **b** the femoral head center as well as **c** 0.5 cm below, **d** 0.5 and **e** 1.0 cm above the femoral head center

**Fig. 3:**
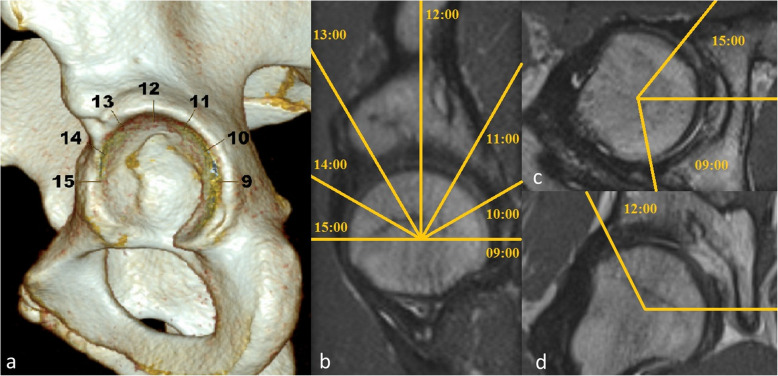
Acetabular sector angles. Measurement of the acetabular sector angle after alignment with the centers of the femoral heads in the transverse and coronal planes: **a** and **b** exemplary with description of all 7 ASA angles (9/10/11/12/13/14/15 o’clock) **c** 9 and 15 o’clock in the transverse plane **d** 12 o’clock in in the coronal plane

**Fig. 4:**
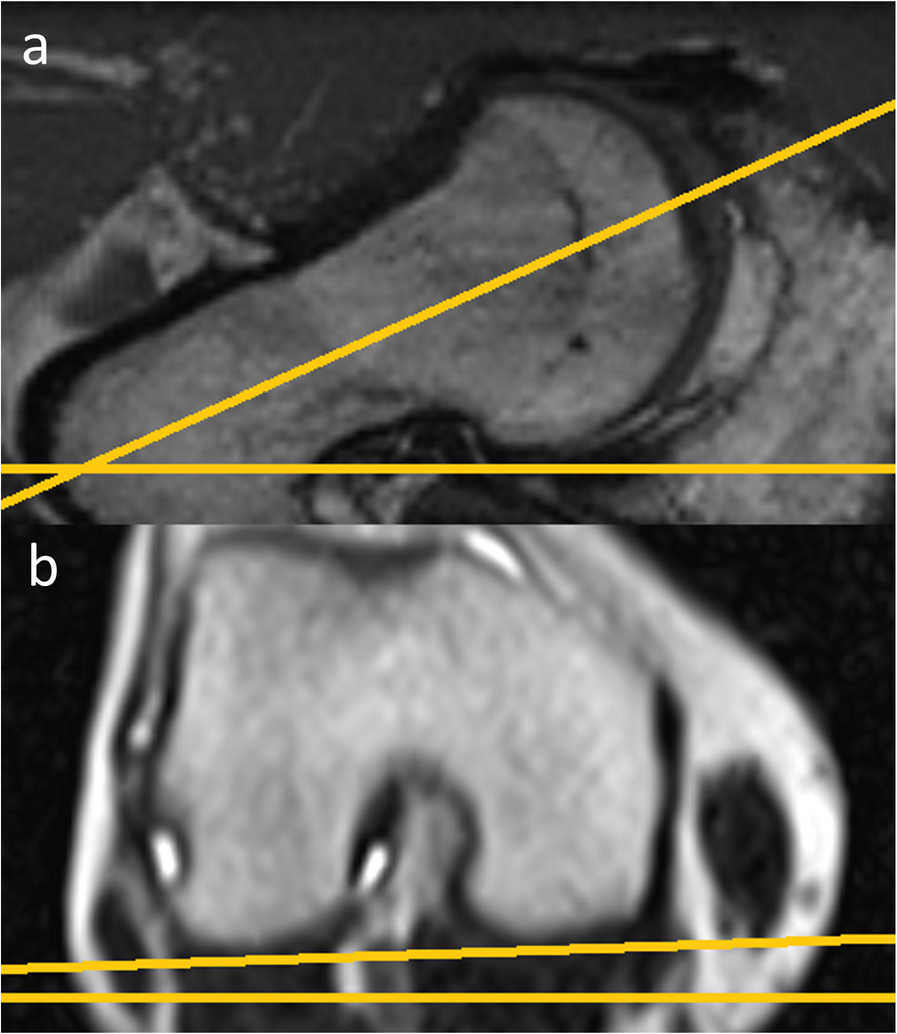
Femoral Torsion. Femoral torsion measurement with **a** an oblique view with a femoral neck bisecting axis at the proximal femur and **b** a tangential axis touching the posterior femur condyles in a transverse plane at the distal femur

**Fig. 5:**
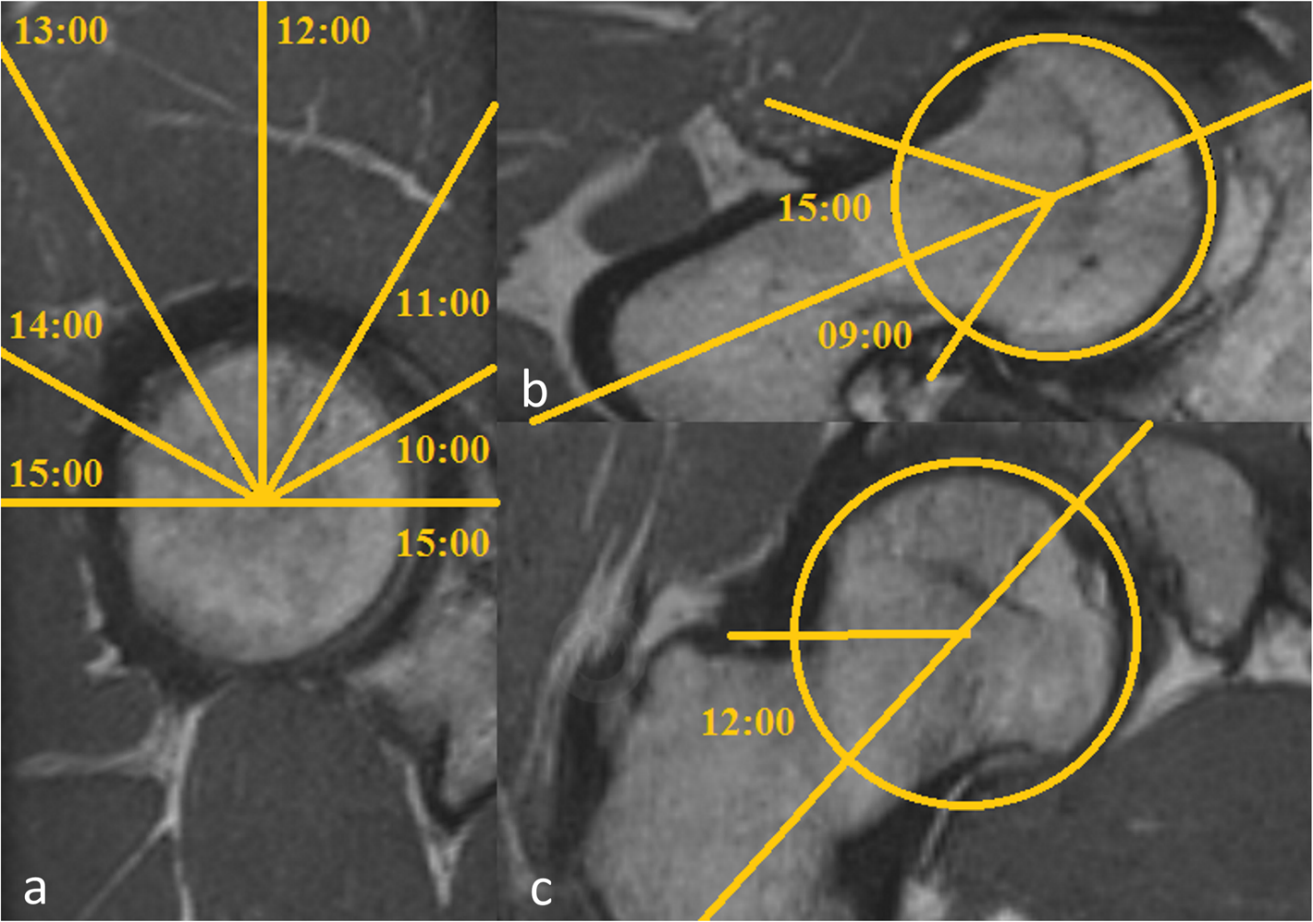
Alpha Angle. Measurement of the alpha angle in a calculated radial sequence using the femoral head center and the femoral neck bisecting line in coronar and axial plane: **a** exemplary with description of all 7 alpha angles (9/10/11/12/13/14/15 o’clock) **b** 9 and 15 o’clock and **c** 12 o’clock

Acetabular morphology was rated by acetabular version at the level of the femoral head center as described by Anda as well as 0.5 cm below, 0.5 and 1.0 cm above the femoral head center (Fig. 2) [13]. In addition seven modified acetabular sector angles (ASA) were measured (Fig. 3a-d) [14]. All ASA were measured in a clockwise position (9/10/11/12/13/14/15 o’clock). 15 o’clock equals the traditional anterior acetabular sector angle (AASA) and 9 o’clock the posterior acetabular sector angle (PASA), respectively, as defined by Anda [15]. The ASA at the 12 o’clock position resembles the center edge angle described by Wiberg subtracted by 90°. Femoral torsion was evaluated in an oblique view with a femoral neck bisecting axis at the proximal femur and a tangential axis touching the posterior femur condyles in a transverse plane at the distal femur (Fig. 4) [16]. Further the McKibbin Index / Combined acetabular and femoral version was calculated. In addition femoral head sphericity at follow-up was assessed by measuring the alpha angle in radial MRI using predefined sectors clockwise from anterior to dorsal (Fig. 5a-c) [17]. Evidence of osteoarthritis of the hip before surgery and progression during follow-up were graded according to the classification system of Kellgren and Lawrence [18]. Assessment of all pre- and postoperative morphologic features of the acetabulum and femoral head was performed by one trained observer (JG).

Statistic was performed using SPSS 23.0 (IBM Statistics, Chicao, Illinois).

To compare groups with normal distribution we used an unpaired t-test. To detect the influence of parameters, we used correlation analysis of Pearson. An error of α 5% was accepted.

## Results

**Fig. 6:**
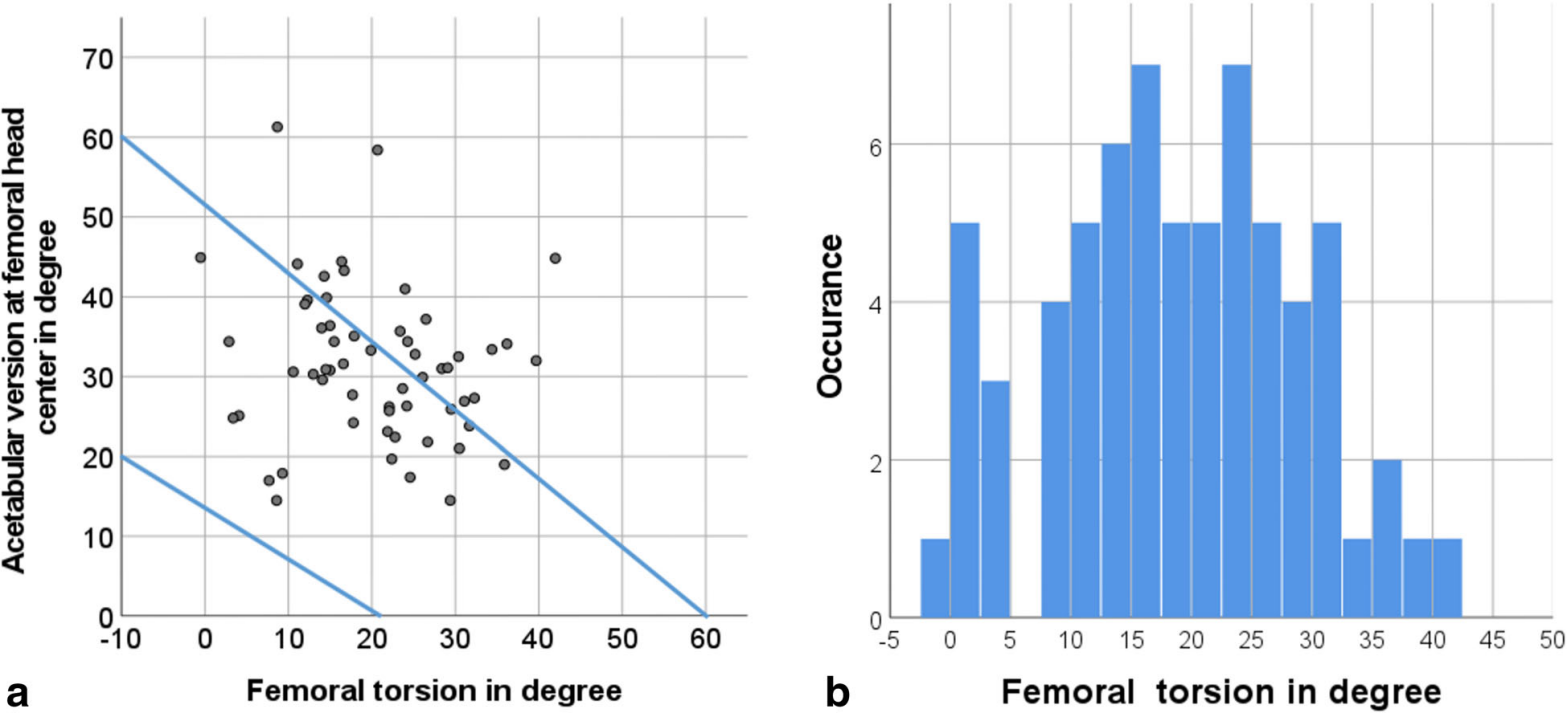
Postoperative Femoral Torsion and McKibbin index. **a** Scatterplot of combined acetabular and femoral version with marked McKibbin index, **b** incidence of femoral torsion in degree with bars of 2.5° presented with absolut numbers

Postoperatively lateral coverage displayed by the 12 o’clock ASA was similar for male (124.4 ± 7.7°) and female (124.9 ± 6.1°) patients. The acetabular version increased overall, but especially for male patients (male 36.6 ± 13.2 vs. female 30.1 ± 8.0) (Table 2). Anterior cover was generally reduced, in particular for male patients at the 15 o’clock ASA (male 24.0 ± 24.5 vs. 37.4 ± 10.7). Altogether the global horizontal coverage by the combined 9 and 15 o’clock ASA was reduced, again especially for male patients (male 117.9 ± 24 vs. female 134.4 ± 11.4). Femoral torsion had a regular distribution for patients with dysplasia (Fig. 6). The calculated McKibbin Index showed no cases of combined anteversion with less than 20° and 21% of the cases had an increased combined anteversion over 60° (Fig. 6). The mean alpha angle in the anterocranial femoral head neck junction was well below 50° (Table 2).

**Table 2:** Radiographic postoperative acetabular und femoral morphology

	Overall	Male	Female
**Acetabular sector Angle**			
15:00	34.7 ± 15.4 (−21.4–64.4)	24.0 ± 24.5 (− 21.4–45.8)	37.4 ± 10.7 (12.2–64.4)
14:00	58.8 ± 20.0 (13.7–104.3)	56.8 ± 5.8 (48.3–68.1)	59.1 ± 22.4 (13.7–104.3)
13:00	115.7 ± 11.3 (53.7–130.2)	117.5 ± 8.6 (100.4–129.9)	115.3 ± 11.8 (53.7–130.2)
12:00	124 ± 6.4 (105.9–138.4)	124.4 ± 7.7 (105.9–132.6)	124.9 ± 6.1 (109.4–138.4)
11:00	123.2 ± 8.6 (98.8–137.5)	123.8 ± 10.2 (98.8–135.9)	123.0 ± 8.3 (100.8–137.5
10:00	112.5 ± 9.8 (76.2–130.5)	116.3 ± 9.1 (101.8–129.3)	111.6 ± 9.8 (76.2–130.5)
09:00	96.7 ± 9.4 (77.5–117.9)	93.9 ± 10.6 (77.5–107)	97.3 ± 9.1 (80.4–117.9)
horizontal (9 + 15)	131 ± 16.1 (73.8–159.1)	117.9 ± 24 (73.8–141)	134.4 ± 11.4 (73.8–159.1)
**Anteversion**			
+ 1,0 cm	29.7 ± 9.2 (5.1–46.5)	30.2 ± 6.2 (18.7–38.6)	29.5 ± 9.9 (5.1–46.50)
+ 0,5 cm	30.0 ± 8.4 (7.8–46.2)	30.6 ± 6.8 (16.6–42.6	29.8 ± 8.9 (7.8–46.2)
Femoral head center	31.4 ± 9.6 (14.5–61.3)	36.6 ± 13.2(17.4–61.3)	30.1 ± 8.0 (14.5–44.9)
-0,5 cm	34.7 ± 11.7 (17.5–62.3)	39.8 ± 14.9 (20.7–62.3)	33.4 ± 10.5 (17.5–59.3)
**Alpha Angle**			
15:00	37.7 ± 10.0 (21.7–81.7)	39.3 ± 5.2 (32.1–47.5)	37.7 ± 10.7 (21.7–81.7)
14:00	37.8 ± 8.2 (25.4–62.2)	38.1 ± 6.3 (29–47.7)	37.7 ± 8.6 (25.4–62.2)
13:00	36.7 ± 8.4 (25.4–91.4)	37.4 ± 3.8 (32.2–45.7)	36.6 ± 9.0 (25.4–91.4)
12:00	36.5 ± 5.2 (24,7-49,6)	41.8 ± 3.7 (34.8–45.6)	35.6 ± 4.8 (24.7–49.6)
11:00	33.7 ± 5.3 (20.9–43.3)	37.3 ± 4.3 (32.5–43.3).	33.0 ± 5.2 (20.9–41.9)
10:00	31.6 ± 5.7 (19.2–50.3)	30.5 ± 6.2 (20.3–40.7)	31.8 ± 5.6 (19.2–50.3)
09:00	32.2 ± 5.7 (21.0–47.2)	29.9 ± 6.6 (22.2–43.9)	32.6 ± 5.5 (21.0–47.2)
**Antetorsion**	19.0 ± 10.3 (− 0.5–42)	15.7 ± 7.2 (3.4–30.4)	19.8 ± 10.8 (− 0,5–42)
**Combined Anteversion**	51.8 ± 12.4 (23.1–86.8)	52.2 ± 14.2 (28.2–79.1)	51.7 ± 12.0 (23.1–86.8)

Table 2 **Postoperative** radiographic angles (Acetabular sector angle, Anteversion, Alpha angle, Antetorsion, Combined antetorsion) in MRI expressed as mean ± SD with range in parentheses in general and for male and female patients

**Fig. 7:**
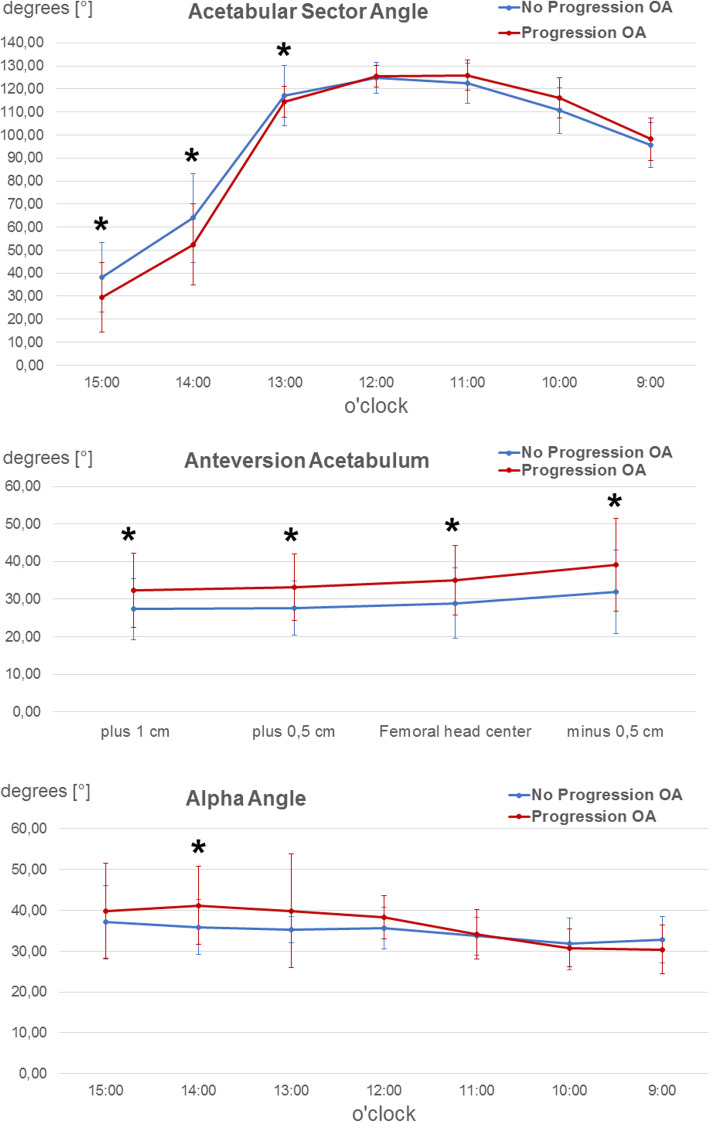
Progression of osteoarthritis and ASA, acetabular version and alpha angle. Illustration of **a** acetabular sector angles, **b** acetabular version and **c** alpha angles in degrees [°] in relation to the acetabular position (9 o’clock to 3 o’clock or acetabular height, as explained in the “Methods” section. The blue curve depicts the hips with no progression of osteoarthritis, the red curve the hips with progression of osteoarthritis. The respective angles are presented as mean with SD (error bars). An asterisk indicates a significant difference (*p* < .05) between the both groups in the respective position

Acetabular morphology in regard to PROMs showed no significant correlation (Table 3). Analyzing cases with progression of osteoarthritis of 1 degree or more defined by Kellgren and Lawrence in comparison to cases with no progression showed a significant heightened acetabular anteversion as well as a reduced acetabular coverage in the anterocranial quadrant (Fig. 7). Yet both groups had similar global acetabular cover horizontally described by the sum of ASA 9 and 15 o’clock (127.6 ± 15.9 vs. 133 ± 16.4; *p* = 0,157). Femoral and combined torsion showed no significant correlation to PROMs or osteoarthritis progression. Comparing patients with a combined anteversion between 20° and 60° and over 60° using the GTO showed no significant difference. The slightly increased alpha angle at the 14 o’clock position had a significant influence on progress of osteoarthritis. At the same time, we detected a significant correlation between an increased alpha angle in the posterocranial quadrant and the GTO as shown in Table 3.

**Table 3:** Correlation of postoperative radiological hip morphologies and functional outcome

	Difference Pre- and Postoperative WOMAC Score	Postoperative Oxford Hip Score	GTO
**Acetabular sector Angle**			
15:00	0.505	0.648	0.854
14:00	0.707	0.234	0.143
13:00	0.247	0.359	0.983
12:00	0.179	0.145	0.140
11:00	0.304	0.258	0.926
10:00	0.939	0.535	0.101
09:00	0.294	0.766	0.134
horizontal 9 + 15	0.224	0.719	0.266
**Anteversion**			
+ 1	0.669	0.227	0.960
+ 0.5	0.390	0.603	0.567
Femoral head center	0.898	0.593	0.706
-0.5	0.641	0.423	0.700
**Alpha Angle**			
15:00	0.080	0.789	0.874
14:00	0.561	0.292	0.702
13:00	0.714	0.916	0.550
12:00	0.153	0.748	**0.470 (0.000)**
11:00	0.077	0.847	**0.259 (0.047)**
10:00	0.162	0.407	**0.308 (0.017)**
09:00	0.979	0.651	0.058
**Antetorsion**	0.342	0.319	0.507
**Combined Anteversion**	0,500	0.835	0.584

Table 3 Correlation analysis between radiographic angles (Acetabular sector angle, Anteversion, Alpha angle, Antetorsion, Combined antetorsion) and PROMs (difference between Pre- and postoperative WOMAC Score, postoperative Oxford Hip score and *GTO* global treatment outcome) with display of the correlation coefficient and if significant *p* value in parentheses

## Discussion

Former studies evaluating PAO correction mostly evaluated the lateral cover of the femoral head as indicator for operation success. Hartig-Andreasen describes a CE angle less than 30° or higher than 40° as a factor for an increased conversion to total hip arthroplasty after PAO [19]. The group around Albers showed results predicting a poor clinical outcome if the postoperative CE angle was below 22° [20]. Steppacher et al. showed similar results using the femoral head extrusion index [3]. Only the group around Beaule did not detect an influence of the CE angle on the postoperative outcome using the WOMAC score as indicator [21]. In our study the ASA at 12 o’clock resembles the measurement position of the CE angle but is not comparable in absolute numbers. We did not detect a correlation between PROMs as well as in osteoarthritis progression in regard to this angle. A reason may be a good postoperative lateral coverage of the femoral head in the majority of all our cases without cases with severe under or over coverage.

The study’s participants showed an increased postoperative acetabular version (male 36.6 ± 13.2°, female 30.1 ± 8.0°) in comparison to healthy patients (male 18 ± 4,5°, female 21 ± 5°). Up to now only two studies analyzed the acetabular orientation after PAO and isolated a decreased acetabular version to be a negative factor for good outcome and progression of osteoarthritis [4, 5]. Since the analysis of the acetabular version can be highly biased by pelvic tilt and other inherent limitations of conventional radiographs we decided to use MRI as a more precise diagnostic tool [6–8]. This makes comparison with other studies more difficult. The reason that we could not detect acetabular retroversion to be a significant factor maybe that we did not have any cases of severe retroverted acetabula with the lowest having an anteversion of 14,5°.

Instead we detected that cases with progression of osteoarthritis had a general larger acetabular anteversion. Patient outcome itself was not influenced by acetabular anteversion.

Further we detected a correlation between reduced anterocranial coverage and progression of osteoarthritis. Since no reference values for the ASA 10 and 11 o’clock exist, it is only possible to compare already established parameters like the anterior ASA at 15 o’clock (AASA) as well as the posterior ASA at 9 o’clock (PASA). In comparison to healthy patients (male: AASA 64 ± 6°, PASA 102 ± 8°; female: AASA 63 ± 6°, PASA 105 ± 8°) both anterior and posterior femoral head cover were still decreased postoperatively [15]. Fuji et al. showed in deformity analysis of not treated dysplasia cases using CT a reduced AASA (male: 42,1 ± 6°, female: 41,3 ± 7,7°) and PASA (male: 84,4 ± 6,2°, female: 91,3 ± 6,8°) and in comparison to a control collective (AASA: 60,7 ± 9°, PASA: 104,5 ± 9,3°) a posterior and anterior undercoverage [22]. Our study population, especially male patients, showed in comparison a reduced AASA as well as a slightly increased PASA in regard to these dysplastic patients. The horizontal ASA showed a global undercoverage for dysplastic hips (male: 117.9 ± 24°, female: 134.4 ± 11.4°) in regard to a healthy collective especially for male patients (male: 167 ± 11°, female: 169 ± 10°) [15]. Since global coverage had no significant influence on patient outcome and progression of osteoarthritis, an uneven anterior/posterior balance of cover i.e. reduced anterocranial cover has possibly more influence on the long-term outcome. The decreased ASAA, especially in comparison to other dysplasia patients’ collectives, as well as the increased acetabular anteversion, suspects an increased version of the acetabular fragment after surgery with a less prominent anterior acetabular rim and cover. Ibrahim et al. analyzed patients with treated cam type deformities and showed that an increased anterior cover is a negative predictor for functional outcome [23]. Since there is a wide coexistence of dysplasia and femoroacetabular impingement [24], which maybe even increases after acetabular correction, a slightly more anteverted placement may be a reasonable orientation of the acetabular fragment. At the same time an exaggerated anteversion can lead to a possible hip instability and progression of osteoarthritis.

Different research groups already established, that a heightened femoral torsion or a retrotorsion may induce osteoarthritis [25, 26]. Research articles evaluating femoral torsion describe different average mean values from 10.4–24.1° for healthy patients [27–29]. Different studies specify that patients with dysplastic hips have an increased antetorsion in comparison to healthy patients [13, 30]. Akiyama et al. describe for dysplastic hip not only an increased anteversion but also a more diverging value for the femoral torsion depending on anterior and posterior coverage of the acetabulum in comparison to healthy patients [31]. Overall, we did not detect a correlation between femoral torsion and PROMs or progression of osteoarthritis after PAO.

The combined acetabular and femoral version, first described by McKibbin 1970, is in our opinion a parameter with increasing relevance [32]. The developed McKibbin index divides the measurements in a group with regular combined version between 20 and 60° as well as below 20° and above 60° with increased incidences of pain. Especially a reduced combined version below 20° is associated with osteoarthritis [33]. Kohno et al. show in a retrospective assessment of 100 dysplastic hips that patients with increased combined anteversion have an early development of pain [9]. Since no patients in our study had a combined anteversion below 20° we could not evaluate these casuistic. In regard to other studies we did not find a difference between patients with a combined version between 20 and 60° and above 60° in regard to pain, PROMs and progress of osteoarthritis.

Cam Impingement and the corresponding increased alpha angle have a significant influence on the outcome after PAO. Beaule et al. describe a correlation between a preoperative increased alpha angle and worse postoperative WOMAC during the follow-up [21]. Albers et al. show in a retrospective follow-up, that patients with normal head/neck ratio had a better outcome as well as less progression of osteoarthritis over 11 years [20]. Since all patients in our study with a preoperative significant heightened alpha angle received an intraoperative femoroplasty, no severe cases of cam impingement occurred postoperatively. Although the group with progression of osteoarthritis showed a significant increased mean alpha angle at 14 o’clock, the alpha angle remained below 50°. Overall, patients with an increased cranial and posterocranial alpha angle (10, 11 and 12 o’clock) had a postoperative decreased GTO. Since the posterocranial head neck junction is the insertion zone for the femoral head vessels a possible correction is difficult. These results reflect that patients with larger head asphericity may have a reduced outcome after PAO. Goronzy et al- showed prospectively in a 5-year-follow-up-study after PAO an equal outcome for patients without cam deformity and surgical correction of the cam deformity [34].

Limitations to our study were the lack of standardized preoperative MRIs for better understanding of preoperative acetabular orientation. Most patients received preoperatively an external MRI which was not comparable with the follow-up MRI with sufficient quality. Still comparison of global acetabular cover (combined ASA) and acetabular orientation amplified by femoral torsion enables us to consequently make a sound conclusion for postoperative placement even without standardized preoperative MRI imaging. Although we had a good follow-up rate of 85.9%, only 66 (66.7%) hip MRIs could be obtained, leaving 33 without 3 dimensional imaging. Control collectives for 3-dimensional measurements in dysplastic hips originate from Asian countries maybe describing a different pelvic morphology and making comparisons difficult. Also, the time period of the follow-up rate of 5 years and the number of MRIs might not be sufficient to asses enough progression of osteoarthritis or decline in PROMs to filter out certain poor placements of the acetabulum.

## Conclusions

In conclusion, only posterior deformed femoral heads had an influence on the functional outcome without alternating degeneration in the 5-year interval. Instead acetabular version and anterior cover had an influence on the progression of osteoarthritis. In addition to known factors such as acetabular retroversion, induced pincer impingement, highly elevated alpha angle we detected decreased anterior coverage and increased acetabular version also to be associated with increased risk of progression of osteoarthritis. For better understanding further studies have to be realized focusing on the long-term outcome. Even though this is a first insight in 3 dimensional orientation of the acetabular fragment after PAO, it is our believe that not only the lateral cover but also the complete acetabular orientation as well as the combined acetabular and femoral version have a significant impact on longterm outcome.

## Data Availability

The datasets during and/or analysed during the current study available from the corresponding author on reasonable request.

## Abbreviations

PAO: Periacetabular osteotomy
MRI: Magnetic Resonance Imaging
WOMAC: Western Ontario and McMaster Universities Osteoarthritis Index
PROMs: Patient reported outcome measures
CE angle: Lateral center-edge Angle
GTO: Global treatment outcome score
SPACE: Sampling Perfection with Application optimized Contrasts using different flip angle Evolution
ASA: Acetabular sector angles
AASA: Anterior acetabular sector angle
PASA: Posterior acetabular sector angle
